# The role of BST‐2/Tetherin in host protection and disease manifestation

**DOI:** 10.1002/iid3.92

**Published:** 2015-12-07

**Authors:** Wadie D. Mahauad‐Fernandez, Chioma M. Okeoma

**Affiliations:** ^1^ Department of MicrobiologyCarver College of MedicineUniversity of IowaIowa CityIA52242USA; ^2^ Interdisciplinary Program in Molecular and Cellular BiologyUniversity of IowaIowa CityIA52242USA

**Keywords:** Antagonists, breast cancer, BST‐2, cancer, HIV‐1, malignancies, tethering, viruses

## Abstract

Host cells respond to viral infections by activating immune response genes that are not only involved in inflammation, but may also predispose cells to cancerous transformation. One such gene is BST‐2, a type II transmembrane protein with a unique topology that endows it tethering and signaling potential. Through this ability to tether and signal, BST‐2 regulates host response to viral infection either by inhibiting release of nascent viral particles or in some models inhibiting viral dissemination. However, despite its antiviral functions, BST‐2 is involved in disease manifestation, a function linked to the ability of BST‐2 to promote cell‐to‐cell interaction. Therefore, modulating BST‐2 expression and/or activity has the potential to influence course of disease.

Early studies performed with monoclonal antibody (anti HM1.24) identified as HM1.24 antigen, that is, today called BST‐2 1. It was then suggested that BST‐2 is expressed in terminally differentiated B cells and may be involved in early pre‐B‐cell development 1. However, emerging evidence suggest that although BST‐2 is broadly expressed in host cells, suppressing BST‐2 levels may be well tolerated as these mice do not present gross phenotypic defects and have no fertility issues compared to BST‐2‐expressing mice 2, 3, 4. Nonetheless, the functions of BST‐2 are still evolving and a more comprehensive study of BST‐2 knockout mice is necessary to better understand cell/tissue type‐dependent functions of BST‐2. The level and expression pattern of BST‐2 is variable, depends on cell or tissue types 4, 5, and can be induced by types I and II interferons, as well as by mitogens, such as lipopolysaccharide (LPS) 4, 6, 7, 8. BST‐2 is primarily located on the apical membrane 9 with some expression present in the trans‐Golgi network (TGN) and in vesicular compartments 10. Endogenously expressed BST‐2 protein contains complex carbohydrate modifications and presents as a smear of multiple 30–40 kDa bands presumed to be due to N‐linked glycosylation 11. In contrast, exogenously expressed BST‐2 is modified by high‐mannose carbohydrates with predicted molecular weight of 28–29 kDa 11.

BST‐2 is a type II transmembrane protein of 180 amino acids 12. Structurally, BST‐2 is composed of an N‐terminal cytoplasmic tail followed by a transmembrane domain (TM), a coiled–coiled ectodomain, and a C‐terminal glycosylphosphatidylinositol (GPI)‐anchor 9 (Fig. 1). The C‐terminal membrane anchor is thought to be a second TM domain rather than a GPI anchor 12. The cytoplasmic tail of BST‐2 contains a highly conserved double tyrosine motif (6**Y**7 × 8**Y**) implicated in clathrin‐dependent endocytosis of BST‐2 13 and in nuclear factor κ‐B (NF‐κB) activation 14, 15, 16, 17 (Fig. 1). The N‐terminal TM domain and the C‐terminal GPI anchor are separated by 120 residues that make up the coiled‐coil ectodomain 18, 19, 20. The N‐terminus of BST‐2 ectodomain comprises of three cysteine residues that are implicated in the formation of covalent cysteine‐linked dimers (home‐dimers and ‐tetramers) 1, 11, 21, 22. The cysteine residues are located at positions 53, 63, and 91 of the human BST‐2 and at positions 58, 68, and 96 of the mouse BST‐2 18. Any of these three cysteines is functional and independently contribute to the formation of cysteine‐linked dimers 11, 22.

**Figure 1:**
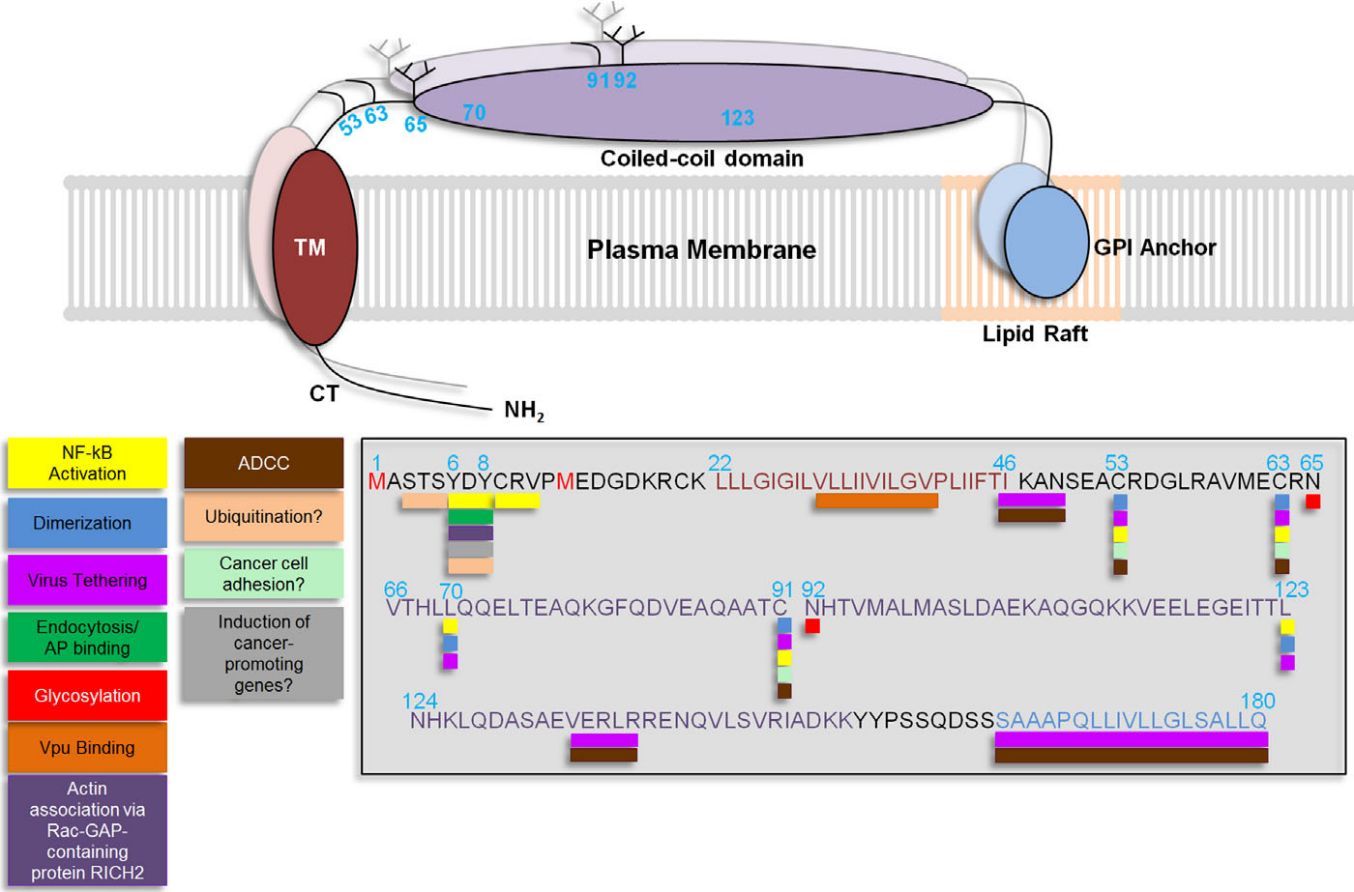
BST‐2 structure: BST‐2 is a type II transmembrane protein with a N‐terminal cytoplasmic tail (CT) followed by a transmembrane domain (TM), a coiled‐coil domain and a glycosylphosphatidylinositol (GPI) anchor embedded in lipid rafts in the cell membrane. The amino acid sequence of BST‐2 depicted in the gray box is color coded with their respective domains. Numbers on top of amino acids correspond to amino acid location. Underneath the amino acid sequences are colored boxes corresponding to different functions and characteristics of BST‐2 including NF‐κB activation (yellow), dimerization (blue), virus tethering (light purple), endocytosis/adaptor protein (AP) binding (green), sites of glycosylation (red), Vpu binding (orange), actin association (purple), motifs for ADCC induction (brown). Also included are some hypothetical characteristics/functions including sites of ubiquitination (light orange), cancer cell adhesion (light green), and induction of cancer‐promoting genes, such as matrix metalloproteinases, CXCR4, CXCL12, and other signaling molecules (gray). BST‐2 contains two translational start sites at methionine 1 and 13 (red) generating a long and short isoform, respectively. The short isoform cannot induce NF‐κB activation since it lacks the YXY motif. BST‐2 forms homo‐dimers and—tetramers through 3 conserved cytosine residues at positions 53, 63 and 91. Leucine residues at positions 70 and 123 are important for maintaining the structure of BST‐2 and for virus tethering, which also requires the C‐terminal GPI anchor.

Additionally, BST‐2 molecules form homo‐tetramers, mediated by leucine residues 70 and 123 that are implicated in promoting proper BST‐2 trafficking 20. Furthermore, BST‐2 ectodomain is post‐translationally modified by N‐linked glycosylation of two asparagine residues at positions 65 and 92 9, 11, 21. Although the function of BST‐2 glycosylation for inhibition of virus release is unclear 11, 22, this post‐translational modification is important for proper folding and trafficking of BST‐2 through the endoplasmic reticulum (ER) and the Golgi 23. BST‐2 molecule associates with lipid rafts 1, 9, 13, 24, 25, 26 through the GPI anchor 9 (Fig. 1). Removal of the anchor does not affect association of BST‐2 with the cell membrane; however, lipid raft localization of BST‐2 is lost 9.

Emerging experimental and clinical evidence on the various functions of BST‐2 and the progress in our understanding of the involvement of innate immune responses to viral infections, inflammation, and cancer has prompted the need for a discussion on the role of BST‐2 in the host. Availability of genetically modified mice and human cell lines has revealed the range of phenotypes associated with BST‐2 in different cells at various physiological and pathophysiological conditions. We start by discussing the role of BST‐2 in viral infections and evolutionary adaptation of viruses to BST‐2, to the new discoveries about the involvement of BST‐2 in disease manifestation. We then describe the various regulatory mechanisms of BST‐2 and by BST‐2, and conclude with perspectives and future possibilities.

## BST‐2/Tetherin: Roles in Viral Pathogenesis

In 2008, BST‐2 was rediscovered as the host factor responsible for preventing the release of HIV‐1 with mutated Vpu (HIV‐1 ΔVpu) from host cells 6, 7. Following these discoveries, BST‐2 was renamed tetherin 7. Since then, the tethering effect of BST‐2 has been shown to extend to other enveloped viruses including rhabdoviruses 27, alphaviruses 28, 29, arenaviruses 30, filoviruses 31, 32, herpesviruses 33, paramyxoviruses 30, orthomyxoviruses 30, 34, orthohepadnaviruses 35, flaviviruses 36, 37, 38, and retroviruses 4, 7, 39, 40 (Table 1). Aside from virus tethering, BST‐2 possesses other antiviral functions and viruses have evolved mechanisms to antagonize BST‐2.

**Table 1:** Viruses susceptible to BST‐2 tethering

Virus	Family
HIV‐1, HIV‐2, SIV, EIAV, MLV, MMTV	Retroviridae
CHIKV, SFV	Togaviridae
Ebola, Marburg	Filoviridae
VSV	Rhabdoviridae
LASV, MACV	Arenaviridae
HSV‐1, HSV‐2, HH‐8	Herpesviridae
Dengue, Hepatitis C viruses	Flaviviridae
HBV	Orthohepadnaviridae
Influenza A virus	Orthomyxoviridae

### Virus tethering

**Figure 2:**
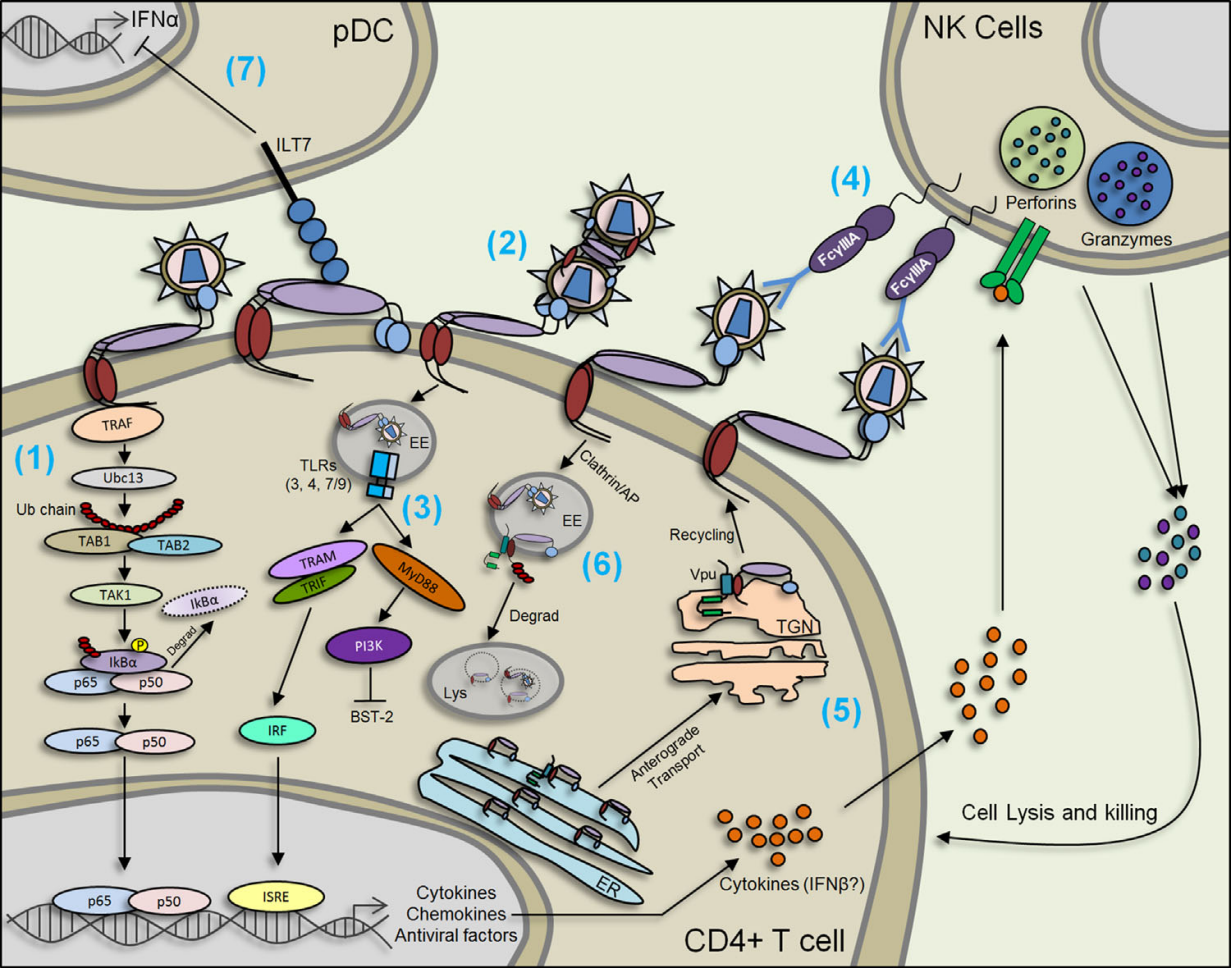
BST‐2 in viral infection: BST‐2 tethers viruses to the cell membrane and to each other (1) and (2). Tethered viruses may be internalized through clathrin coated pits mediated by the YXY motif on BST‐2's cytoplasmic tail (CT). Virus tethering by BST‐2 also induces the activation of NF‐κB through BST‐2's YXY motif which interacts with TRAF and requires Ubc13, Tab1, Tab2, and TAK1 (1). NF‐κB activation induces the expression of cytokines, such as CXCL10 and Il‐6 and may induce the expression of BST‐2 since the BST‐2 promoter contains elements for NF‐κB binding (1). In the early endosome (EE), TLRs recognize different viral factors and induce the expression of cytokines, chemokines and several antiviral factors that contain IFN‐sensitive response elements (ISRE) on their promoter (3). Signaling through TLRs regulates BST‐2 in a positive and/or a negative manner (3). BST‐2 tethering is also required for the induction of antibody‐dependent cellular cytotoxicity (ADCC) which in turn induces the degranulation of effector cells, such as natural killer (NK) cells, through the FcγIIIA receptors (4). Viruses have evolved mechanisms to antagonize BST‐2. The best studied BST‐2 antagonist is Vpu which interacts with BST‐2 at the endoplasmic reticulum (ER), trans‐Golgi network (TGN) and early endosomes. Vpu sequesters BST‐2 at all of these sites. Vpu can also prevent anterograde transport of BST‐2 from the ER to the TGN, and prevent BST‐2 recycling from TGN to the plasma membrane (5). Vpu also promotes BST‐2 degradation (Degrad) through the lysosomal pathway (Lys = lysosome) (6). BST‐2 is a ligand for ILT7 (immunoglobulin‐like transcript 7) at least in plasmacytoid dendritic (pDC) cells. Engagement of ILT7 by BST‐2 represses IFNα expression which in turn can induce BST‐2 levels (7).

The unique topology of BST‐2 (Fig. 1) allows it to tether enveloped viruses to the surface of infected cells 9, 41. One of the structural arrangements that facilitates efficient virion tethering by BST‐2 is one in which the GPI anchor of cell‐associated BST‐2 inserts into the viral membrane of budding virus 22, 41 as the cytoplasmic tail of BST‐2 is necessary to initiate intracellular signaling cascades (Fig. 2 #1). However, structures in which the transmembrane domain inserts into the viral membrane is plausible 42. Also possible is an arrangement in which the entire BST‐2 protein buds along with the virus 22, 43, 44 (Fig. 2 #2). Virus tethering by BST‐2 is mediated in part by the ability of BST‐2 to form homo‐dimers through covalent bonds between cysteine residues in the ectodomain of BST‐2 42. By tethering enveloped viruses to the surface of infected cells, BST‐2 not only restricts virus release but it also elicits and amplifies innate immune responses through the induction of cytokine/chemokine expression 14, 45, a process believed to largely involve BST‐2 cytoplasmic tail. Indeed, the Y × Y motif on BST‐2 cytoplasmic tail is implicated in NF‐κB activation involving recruitment of TAK1, Ubc13, TRAF2, and TRAF6 14, 46 (Fig. 2 #1). Moreover, BST‐2 associates with the cortical actin cytoskeleton through the Rac‐GAP‐containing protein RICH2 (Fig. 1) and abrogation of this interaction significantly diminishes NF‐κB activation 47. In virus infected cells, the cortical actin cytoskeleton mediates BST‐2 phosphorylation and recognition of the YXY motif by the spleen tyrosine kinase (Syk) and subsequent NF‐κB activation, culminating in expression of CXCL10 and IL‐6 47. Additionally, NF‐κB activation by BST‐2 is not only mediated by virus tethering but can also result from antibody crosslinking 14, suggesting that BST‐2‐induced signaling and cytokine/chemokine production could result from a variety of stimuli.

NF‐κB is a promiscuous transcription factor that regulates the expression of several cytokines and chemokines 14. It is still contested whether internalized or cell membrane‐associated BST‐2 mediates NF‐κB activation 14, 46. Following virus tethering, BST‐2 facilitates virus internalization to early endosomes and subsequent lysosomal degradation. The resulting viral products serve as PAMPs that activate TLRs 6, 48, 49 (Fig. 2 #3). Interestingly, TLR4 positively regulates BST‐2 expression 8; a scenario that may lead to a feedback loop following recognition of PAMPs by TLRs, as well as activation of signal transduction cascade capable of inhibiting viral replication or possibly promoting replication if enhancing factors are induced.

Aside from inhibiting viral clearance through activation of signal transduction pathways, tethered viruses may regulate cell‐to‐cell viral spread. BST‐2 may enhance cell‐to‐cell viral spread by the formation of viral clusters 50. However, inhibition of cell‐to‐cell viral spread by BST‐2 could occur by initiation of virological synapses, by trapping viruses in intracellular compartments 51, 52, 53, or by eliciting antibody‐dependent cellular cytotoxicity (ADCC) 54, 55, 56 (Fig. 2 #4). Indeed, the Env of tethered viruses may contain epitopes recognized by cytotoxic‐inducing antibodies (Abs). Binding of these Abs to Env leads to degranulation of effector cells, such as NK cells via FCγIIIA receptors 56, resulting in the release of perforins and granzymes, that may lyse and kill infected cells, respectively 57. Although no primary data exist in support of ADCC‐mediated enhancement of infection, it is noteworthy that ADCC‐mediated cell lysis may result in the release of viral particles that are tethered or trapped in intracellular compartments resulting in viral spread. Further research is needed to better understand the phenomenon of ADCC and in BST‐2‐mediated antiviral activities.

### Inhibition of virus replication

The role of BST‐2 in the replication of various viral families is beginning to emerge, albeit slowly. In mouse model of alphavirus infection, BST‐2 potently inhibits Chikungunya virus (CHIKV) infection and viral replication. BST‐2 deficiency increases viral load at the inoculation site, enhances plasma viremia and lymphoid tissues viral tropism 45. Moreover, BST‐2 deficiency impairs CHIKV‐induced inflammatory response that manifests as reduced levels of IFN‐α, IFN‐γ, and CD40 ligand 45. Aside from its role in alphavirus replication, BST‐2 inhibits replication of retroviruses including MMTV and MLV in mice 3, 4, 58. Inhibition of retrovirus replication is thought to be partly the result of endocytosed BST‐2‐mediated induction of IFNγ production and degranulation of effector cells (NK and CD8+ T cells) 58. Interestingly, endocytosis‐defective BST‐2, which is highly concentrated on cell membranes, is less efficient in restricting viral spread compared to an endocytosis‐competent BST‐2 58. These data suggest that while the tetherin function of BST‐2 is important, virus tethering may be dispensable or play a sensing role in the induction of immune response and BST‐2‐mediated inhibition of virus replication in vivo.

## Viral Antagonists of BST‐2 and Neutralization of BST‐2 Antiviral Function

Different viral proteins antagonize BST‐2. The mechanisms of BST‐2 antagonism by these viral proteins vary and include protein trapping in intracellular compartments, proteasomal and lysosomal degradation of BST‐2, inhibition of BST‐2 anterograde transport, inhibition of recycling, and other yet to be identified mechanisms. In the following section, we discuss viral antagonists of BST‐2 and the known or putative mechanisms of action.

### HIV‐1 Vpu

HIV‐1 Viral protein U (Vpu) 59, 60 is renowned for its role in proteasomal degradation of CD4 61, 62 and enhancement of HIV‐1 release from infected cells 63, 64, 65 in a cell type‐dependent manner 66. In 2008, two independent laboratories showed that cell type‐specific expression of BST‐2 correlates with Vpu‐dependent release of HIV‐1. Suppression of endogenous BST‐2 expression resulted in Vpu‐independent virus release whereas rescue of expression with exogenous BST‐2 in cells that otherwise do not have high BST‐2 renders these cells Vpu‐dependent for virus release 6, 7. These findings revealed that BST‐2 is an inhibitor of virus release and a target of Vpu 6, 7. These observations gave credence to an earlier study in 2006 that showed that expression of Vpu‐reduced BST‐2 levels in HeLa cells 67, thus providing a functional association between Vpu and BST‐2. The interaction between Vpu and BST‐2 requires the transmembrane domain of both Vpu 68, 69 and BST‐2 70, 71, 72 (Fig. 1). To neutralize the effect of BST‐2, Vpu utilizes various mechanisms as discussed below.

### Enhancement of BST‐2 degradation

Vpu promotes intracellular down‐regulation of BST‐2 73, 74, a mechanism that involves beta‐transducin repeat containing protein 2 (β‐TrCP2) 75, 76. β‐TrCP2 is an E3 ligase that forms the SCF^β‐TrCP2^ complex involved in lysosomal degradation 77. Phosphorylation of serine residues at positions 52 and 56 (catalyzed by casein kinase II (CK2) of Vpu is critical for the interaction of Vpu with β‐TrCP2 and for BST‐2 degradation 78, 79. Following Vpu and β‐TrCP2 interaction, the latter interacts with E3 ligase core component Cullin1 (Cul1) through the S‐phase kinase associated protein 1 (Skp1) forming the SCFβ‐TrCP2 complex. Cul1 then associates with ring‐box protein 1 (Rbx1) mediating Cul1 neddylation (addition of an ubiquitin‐like NEDD8 moiety), which changes the conformation of Cul1 allowing recruitment of specific E2 enzymes. Generally, the SCF^β‐TrCP2^ complex ubiquitinates substrates bound to β‐TrCP2 80. However, in HIV‐1 infected cells, Vpu acts as an adaptor to facilitate β‐TrCP2 recruitment and BST‐2 ubiquitination 79. How Vpu commits BST‐2 to lysosomal degradation and avoids a similar fate is unknown. Possibly, Vpu dissociates from BST‐2 and β‐TrCP2 in the early endosome after endosomal sorting complex required for transport‐0 (ESCRT‐0) recognizes and ubiquitinates BST‐2. ESCRT‐0 acts as a checkpoint to commit a protein for lysosomal degradation and it is essential for BST‐2 degradation by Vpu 81, 82. ESCRT‐0 contains two subunits—signal transducing adaptor molecule 1 (STAM1) and hepatocyte growth factor‐regulated tyrosine kinase substrate (HRS) 83. These subunits contain two ubiquitin binding domains (UBDs)—UIM (ubiquitin interacting motif) and VHS (Vps27/Hrs/STAM) 84. The domain(s) necessary for ubiquitinated BST‐2 recognition and degradation are yet to be identified.

Although much of the mechanistic details of this interaction have been resolved, there is no consensus on specific residues on BST‐2 cytoplasmic tail that are ubiquitinated and the ubiquitin topology involved in BST‐2 degradation is unknown (Fig. 1). As the SCF^β‐TrCP2^ complex leads to lysosomal degradation, the K63 topology of the ubiquitin chain may be involved. Despite all the evidence supporting the role of Vpu in neutralizing BST‐2‐mediated tethering of HIV‐1 particles and subsequent viral spread, loss of Vpu does not completely prevent cell‐free HIV‐1 dissemination but pushes the mechanism of virus dissemination toward a cell‐to‐cell‐based mode. Moreover, primary HIV‐1 isolates in which Vpu harbors a start codon mutation are capable of disseminating in vivo 85, 86. Therefore, the role of BST‐2 and Vpu in HIV‐1 infection and/or host response to HIV‐1 still remains unresolved.

### Inhibition of anterograde transport

Vpu and BST‐2 are both present in early endosomes 87. The close cellular localization of Vpu and BST‐2 suggest that Vpu may either recruit the SCF^β‐TrCP2^ complex to ubiquitinate BST‐2 and commit it to lysosomal degradation 23, 88 as discussed above or shuttle BST‐2 to the TGN for possible degradation. Experimental evidence suggest that Vpu does not affect BST‐2 internalization, but rather prevents proper intracellular transportation of newly synthesized BST‐2 from the ER and TGN to the cell membrane 89, 90 (Fig. 2 #5) and/or from early endosomes to the cell surface 44, 88, 91 (Fig. 2 #6). Vpu‐mediated inhibition of BST‐2 anterograde transport occurs in the absence of Ser52 and Ser56 phosphorylation 89, 90, 91.

### Removal from lipid rafts

Studies that examined the activity of certain Vpu mutants revealed that surface down‐regulation and/or degradation of BST‐2 are not enough to explain Vpu‐mediated enhancement of virion release 73. HIV‐1 preferentially buds from areas of the cell membrane that contains lipid rafts where BST‐2 C‐terminal GPI anchor is embedded. Indeed, the GPI anchor is incorporated into newly formed viral membrane as the virus buds 22, 43. To remove BST‐2 from lipid rafts, Vpu forms a complex with BST‐2 through a tryptophan residue at position 76 (Trp‐76) located on the cytoplasmic tail of Vpu that functions to anchor Vpu C terminus to the lipid bilayer, thus displacing BST‐2 from virion‐assembly sites, while maintaining the levels of BST‐2 in the cell 92.

### Inhibition of antibody‐dependent cellular cytotoxicity (ADCC)

Vpu potently counteracts BST‐2‐mediated ADCC activity 54, 55 via a mechanism that is not clearly understood (Fig. 2 #4). Available data suggest that Vpu prevents BST‐2‐mediated ADCC by trapping BST‐2 in intracellular compartments and that Vpu‐mediated degradation of BST‐2 is dispensable 55.

## Viral Glycoproteins and BST‐2 Neutralization

Neutralization of BST‐2 by the envelope glycoprotein (gp41) of HIV‐2 (HIV‐2 Env) occurs through sequestration of BST‐2 in perinuclear compartments, most likely at the TGN 93 in the absence of BST‐2 degradation. This Env‐mediated neutralization of BST‐2 effect is similarly to the antagonistic actions of Vpu 93, 94 and as reported earlier for Vpu in promoting viral particle release 95, 96, 97, 98, 99. Env interacts with BST‐2 but the domains involved in the interaction are yet to be identified. However, the tyrosine‐based endocytic motif GYxxθ on Env cytoplasmic tail binds AP‐2 allowing clathrin‐mediated endocytosis, required for BST‐2 downregulation from the cell surface 94, 99, 100. The host GTPase dynamin 2 that pinches off clathrin‐ and non‐clathrin‐coated vesicles is involved in HIV Env‐mediated antagonistic effect on BST‐2 100. In addition, the envelope proteins from other lentiviruses, such as SIV or EIAV are known BST‐2 antagonists because their presence enhances viral release in cultured cells 40.

Aside from lentiviral Env, other viral glycoproteins, such as Ebola (Ebo GP) and herpesviruses (HSV) glycoproteins neutralize BST‐2‐mediated tethering. Ebola GP antagonizes BST‐2 tethering function without removing BST‐2 from lipid rafts 31. In the presence of Ebo GP surface BST‐2 protein is greatly reduced 32 without affecting total protein levels, suggesting that GP may downregulate BST‐2 from the cell surface. Considering the findings from both studies 31, 32, a model where Ebo GP internalizes BST‐2 in its lipid raft complex can be envisioned. In addition, GP prevents the interaction of BST‐2 with Ebola VP40 (viral matrix protein) which may prevent virus tethering 101. Despite its ability to tether Ebola particles, BST‐2 does not inhibit Ebola replication 102.

BST‐2‐mediated tethering of *Herpesviridae* family of viruses is controversial. BST‐2 tethers γ‐herpesvirus—KSHV 103, and α‐herpesviruses—herpes virus simplex 1 and 2 (HSV‐1 and HSV‐2) 104, 105 and BST‐2 incorporates into HSV‐2 virions 104. However, BST‐2 does not tether the β‐herpesvirus—human cytomegalovirus (HCMV). Rather, it was reported that BST‐2 enhanced HCMV entry into host cells 106. Similar to HIV, the tethering functions of BST‐2 on HSV‐1 and HSV‐2 is neutralized by various viral products. HSV‐1 glycoprotein gM but not gB and gD neutralizes BST‐2 tethering 105. In contrast, HSV‐2 glycoproteins gB, gD, gH, gL but not gE, gG, or gM reduces the levels of BST‐2 via unknown mechanisms 104.

Other viral glycoproteins of interest are the Sendai virus (SV), fusion (F), and hemagglutinin‐neuraminidase (HN). These SV glycoproteins synergistically neutralize BST‐2 by mechanisms that may involve BST‐2 degradation 107. It has recently been shown that BST‐2 tethers hepatitis B virus (HBV) and that HBV antagonizes BST‐2 108. The tethering function of BST‐2 is also neutralized by hepatitis B virus (HBV) surface protein (HBs). The mechanism of neutralization is thought to involve the ability of HBs to bind BST‐2 and prevents BST‐2 homodimerization 35.

## Antagonism of BST‐2 by HIV‐2 and SIV Negative Regulatory Factor (Nef)

Nef is a 27‐35 kDa myristoylated protein encoded by human and simian immunodeficiency viruses; HIV and SIV. Interaction of BST‐2 and Nef occurs through association of BST‐2 cytoplasmic tail with residues in the Nef N‐terminus that interacts with AP‐2 proteins involved in clathrin‐mediated endocytosis 109, 110, 111. Although the precise mechanism of BST‐2 neutralization by Nef is unknown, it is possible that Nef uses the lysosomal pathway similar to that used in degradation of MHC class I and CD4 112, 113 to degrade BST‐2 109.

## Herpesvirus 8 K3 and K5‐Mediated Neutralization of BST‐2

Herpesvirus 8 also known as Kaposi sarcoma‐associated herpesvirus (KSHV) contains viral factors, K3/MIR1 and K5/MIR2. These proteins are part of the RING‐CH (MARCH) ubiquitin ligase family and are involve in the proteasomal degradation of several antiviral factors including MHC class I receptors, B7‐2, CD166, CD31, ICAM‐1, and BST‐2 114. K3 and K5 ubiquitinate lysine residues located on BST‐2 cytoplasmic tail as BST‐2 is processed out of the ER resulting to the proteasomal degradation of BST‐2 and enhanced KSHV release 103, 115.

## Chikungunya Virus Nonstructural Protein 1 (CHIKV nsP1) Antagonizes BST‐2

CHIKV and Semliki Forest virus (SFV) are two alphaviruses that are susceptible to BST‐2 tethering effect 28, 29, 45. Of all CHIKV envelope proteins (E1, E2, and E3) and non‐structural proteins (nsP1, nsP2, nsP3, and nsP4), only E1 and nsP1 co‐localize with BST‐2. However, only nsP1 overcomes BST‐2‐mediated tethering and enhances CHIKV release through unknown mechanisms 28.

## Influenza Neuraminidases Neutralizes BST‐2

In cultured cells, influenza neuraminidase (N) N1 and N2 antagonize the effects of BST‐2 and rescue influenza release through a yet to be determined mechanism 34, 116. Influenza nonstructural protein 1 (NS1) also antagonizes BST‐2 by averting IFN signaling and infection with this virus results in loss of BST‐2 steady state levels 117. Contrary to the report on the susceptibility of influenza virus to BST‐2‐mediated tethering, a study suggests that BST‐2 does not tether influenza virus and influenza neuraminidase, hemagglutinin, and NS1 are unable to neutralize BST‐2 118.

## BST‐2/Tetherin: Roles in Carcinogenesis

Despite all we have learnt about the antiviral functions of BST‐2 and evolutionary adaptation of viruses to this protein, intriguing new discoveries about the involvement of BST‐2 in carcinogenesis has opened another world of possibilities for BST‐2 biology and function.

The spectrum of BST‐2 expression in various cancers has been revealed using meta analyses studies of large tumor datasets 119. In solid tumors, BST‐2 expression is elevated in head and neck cancer 120, lung cancer 121, breast cancer 119, 122, 123, cervical cancer 124, myelomas 125, 126, endometrial cancer 127, and glioblastoma 128. In addition, data from proteinatlas.org reveal that BST‐2 is overexpressed in colorectal cancer, ovarian cancer, thyroid cancer, and pancreatic cancer (http://www.proteinatlas.org/ENSG00000130303-BST2/cancer). The significance of elevated BST‐2 in various cancers is beginning to evolve. However, not all cancers have elevated BST‐2 119 (Table 2). Compared to normal tissues, BST‐2 expression in lung adenocarcinoma and thyroid cancer is unchanged 119 whereas levels of BST‐2 in lung squamous cell carcinoma, kidney papillary cell carcinoma, kidney chromophobe carcinoma, liver, and prostate cancer is significantly downregulated 119. Thus, in some cancers, constitutive upregulation of BST‐2 expression and BST‐2 activity correlates with disease pathology in human and have been functionally demonstrated to cause disease in mouse models of breast cancer.

**Table 2:** BST‐2 mRNA profile in different cancers

	Profile of BST‐2 transcript between normal and tumor tissue		
Cancer type	Unchanged	Suppressed	Elevated
Lung adenocarcinoma	X		
Thyroid cancer	X		
Lung squamous cell carcinoma		X	
Kidney papillary cell carcinoma		X	
Kidney chromophobe carcinoma		X	
Liver cancer		X	
Prostate cancer		X	
Head and neck cancer			X
Lung cancer			X
Breast cancer			X
Cervical cancer			X
Myelomas			X
Endometrial cancer			X
Glioblastoma			X

### Functional roles of BST‐2 in cancer

Correlation studies using meta analyses of various tumor datasets showed that BST‐2 levels are proportional to the aggressiveness of different cancers including breast 123, 129, brain 128, and oral cavity cancers 120. In vitro, overexpression of BST‐2 in breast cancer cells enhanced cell migration, invasion, proliferation, and anchorage‐independent growth 119, 123, 129 whereas BST‐2 suppression results in reduced migration, invasion, anchorage‐independent growth but not cell proliferation 45. In contrast to the effects of BST‐2 on breast cancer cells, in HT1080 (human fibrosarcoma epithelial cell line) and MDCK (canine kidney cells) cells, overexpression of BST‐2 decreased cell growth and migration due to reduced matrix metalloproteinase 2 (MMP‐2) activity 130. Differences in BST‐2 effect on cancer cells could be due to the cell types used.

Despite the contradictory effects of BST‐2 in various cancers in vitro, Sayeed et. al., showed that elevated BST‐2 expression renders high grade breast cancer cells resistant to pro‐apoptotic drug (tamoxifen and staurosporine) treatment 123. These data point to a functional role of BST‐2 in breast cancer both in the promotion/progression of breast cancer and its resistance to treatments. The Okeoma group used two syngeneic metastatic breast cancer models to demonstrate that BST‐2 plays a functional role in driving breast cancer in vivo 122. Mice injected with metastatic breast cancer cell lines in which BST‐2 was downregulated showed decreased tumor growth at the primary and metastatic sites with resultant increase in survival of tumor bearing mice 131. Although elevated BST‐2 expression enhanced tumor growth, there was no correlation between tumor growth at the primary and secondary sites; suggesting that BST‐2 effects on primary tumor growth are independent of its effects on metastatic tumor growth 131.

**Figure 3:**
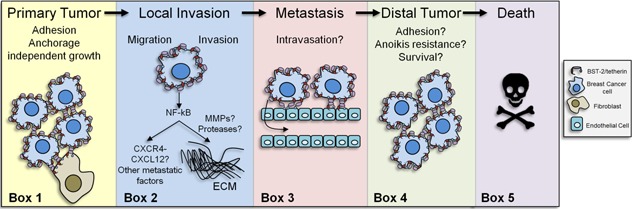
BST‐2 in cancer: BST‐2 is overexpressed in several cancers including breast cancer. In a murine breast cancer model, BST‐2 was found to enhance the adhesion of cancer cells to fibroblasts and may also mediate the adhesion between cancer cells and between other cells found in the tumor microenvironment, such as endothelial cells or macrophages. This ability of BST‐2 may be involved in primary and secondary (distal) tumor formation (Box 1). Once a primary tumor forms, some cells acquire an invasive phenotype a step that may also involve BST‐2 via its ability to induce NF‐κB activation. Through its YXY motif, BST‐2 may induce the expression of metastatic factors, such as CXCR4 (C‐X‐C chemokine receptor type 4) and its ligand CXCL12 (C‐X‐C motif chemokine 12) or invasive factors such as matrix metalloproteinases (MMPs) or proteases that degrade extracellular matrix (ECM) proteins (Box 2). Following invasion through the ECM, cancer cells metastasize to distal sites after intravasation. BST‐2 is involved in monocyte adhesion to endothelial cells and may also allow cancer cell adhesion to endothelial cells prior to reaching blood vessels (Box 3). Metastatic cells must survive in circulation and become resistant to anoikis. Thus, BST‐2 may render cancer cells resistance to anoikis and may enhance cell clustering and cell survival in circulation. To colonize secondary sites, cancer cells must adhere to ECM. BST‐2 mediates cancer cell adhesion to fibronectin and collagen, two well‐known ECM proteins. BST‐2 also enhances cancer cell to cancer cell adhesion. These processes may promote survival by associating cancer cells to each other (Box 4). The increase in tumor growth and metastasis due to high BST‐2 levels results in decreased host survival (Box 5). The gray box to the right is the key.

The molecular mechanism by which BST‐2 promotes cancer remains to be determined. BST‐2 dimerization mediated by three cysteine residues located in the ectodomain is required for viral lipid membrane association with the host membrane. It is possible that BST‐2 uses this mechanism to “tether” cells to each other thereby promoting cell‐to‐cell interaction. Indeed, BST‐2 mediates adhesion of monocytes to endothelial cells 132. The ability of BST‐2 to mediate adhesion was also demonstrated in breast cancer cells where suppression of BST‐2 significantly decreased cell‐to‐cell interaction as well as cell to extracellular matrix (ECM) interactions with collagen and fibronectin 122 (Fig. 3, Box 1). The significance of BST‐2 to facilitate cell adhesion is under investigation. However, cell–cell interactions between cancer cells and stromal cells or between cancer cells and ECM facilitate tumor growth at the primary and distal sites. The ubiquitous presence of BST‐2 in breast cancer cells and the enhanced cell adhesion of these cells suggest effect on tumor growth. Mahauad‐Fernandez et al., 2014 revealed that cancer cells with suppressed BST‐2 are defective in the formation of primary and metastatic tumors and that growth of BST‐2‐suppressed cells in agar (colonies) was diminished compared to high BST‐2 expressing cells 122. In colony formation assay, tumor colonies result from the growth of single cells independent of attachment to plastic. However, cells have to associate with each other and BST‐2 appears to promote this association.

Another plausible mechanism by which BST‐2 may promote cancer development and progression is through activating NF‐κB‐mediated signal transduction pathways. We posit that BST‐2‐mediated cell‐to‐cell adhesion (that mimics BST‐2‐mediated virus tethering) is the mechanism of NF‐κB activation. BST‐2‐mediated activation of NF‐κB 14, 15, 16, 17 may result in the induction of several factors involved in cancer cell migration and invasion including matrix metalloproteases, chemokines, or growth factors (Fig. 3, Box 2), as well as in intravasation of tumor cells (Fig. 3, Box 3), and resistance to anoikis 133, 134 (Fig. 3, Box 4). Anoikis is a detachment‐induced cell death and normal adherent cells with low BST‐2 undergo anoikis in the absence of anchor. The ability of BST‐2‐expressing cancer cells to grow and form colonies independent of anchor positively correlates with anoikis resistance. In this case, the outcome of elevated BST‐2 in breast cancer is poor survival (Fig. 3, Box 5) as revealed by meta analyses of large human datasets 123, 131 and experimental evidence in mouse models of breast cancer 131.

## BST‐2 as a Therapeutic Target for Cancer Immunotherapy

Multiple myeloma (MM) is a type of blood cancer in which plasma cells become malignant. MM is characterized by elevated BST‐2 (HM1.24) expression in malignant plasma cells and in MM cancer stem cells (CSC). Monoclonal antibodies (mAb) against BST‐2 have been used for radioimmunodetection of human MM xenografts 135. These antibodies induced antibody‐mediated cellular cytotoxicity and cytotoxic T cell (CTL) responses against MM cells 136, resulting in MM CSC elimination 137. Similar to MM, anti‐BST‐2 mAb was used as treatment for lung cancer. The treatment elicited ADCC and other complement‐dependent cytotoxicity (CDC) in lung cancer cells 121, 125, 138. The anti‐BST‐2 mAb‐mediated ADCC effect on lung cancer cells was enhanced following treatment with IFNβ and IFNγ 121. In a renal cell carcinoma xenograft model, IFN‐induced BST‐2 enhanced anti‐BST‐2 mAb‐mediated ADCC 139. Additionally, mAb against BST‐2 induced ADCC and CDC in BST‐2 positive endometrial cancer cells in vitro and tumor growth inhibition was achieved in a xenograft model 127.

Although some success with antibody against BST‐2 on some cancers was achieved, an experiment with orthotopic mouse brain tumor model (using GL261 brain tumor cells) was unsuccessful 128. Even though the levels or BST‐2 were high in these brain tumor cells, there was no therapeutic significance following RNAi‐mediated downregulation of BST‐2 or pretreatment of cells with anti‐BST‐2 mAb 128.

In virus infected cells, antibody against BST‐2 enhances virus release by redistributing and removing BST‐2 from the sites of virus budding 140. It is possible that antibody cross‐linking with BST‐2 may change BST‐2 localization and enhance BST‐2‐mediated signaling 14, 140. With these in mind, it remains to be determined whether anti‐BST‐2 mAb‐based immunotherapy has a long term therapeutic effect on the cancers discussed above as well as on other solid cancers. As at the time of this review, no other BST‐2‐based therapeutic attempts have been made.

## Other Roles That BST‐2/Tetherin Plays

Various other functions have been associated with BST‐2. BST‐2 plays a role in regulating the development of regulatory T (Treg) cells in the thymus 141. BST‐2 also regulates autophagy by interacting with the autophagy/mitophagy suppressor LRPPRC and preventing LRPPRC from binding to Beclin 1 and the anti‐apoptotic protein Bcl‐2. This interaction abrogates binding of Beclin 1 to PI3KCIII, thus initiating autophagy 142. A caveat is that these experiments were performed in transformed HeLa and 293T cells. Whether BST‐2 induces autophagy in immune cells that are relevant to most virus infection is yet to be determined. BST‐2 in guinea pig is necessary for the maintenance of Golgi integrity and function 143. Moreover, BST‐2 is important for the organization of membrane micro‐domains. BST‐2 plays a role in organization of lipids in the plasma membrane and in the distribution of proteins that are confined to lipid rafts 144. All domains of BST‐2 are important for this function as opposed to other functions of BST‐2 such as virus tethering in which the cytoplasmic tail of BST‐2 is dispensable. This suggests that BST‐2's ability to homo‐dimerize or tetramerize 87 and its ability to interact with the actin cytoskeleton 47 are essential for its micro‐domain organizing function.

## BST‐2 Regulation

### Interferon (IFN)‐mediated BST‐2 regulation

IFNs play important roles in host defense against viral infection by inducing the expression of a diverse range of antiviral factors, including BST‐2. In various cells, BST‐2 is induced by type I IFN (IFNα and IFNβ), type II IFN (IFNγ), and type III IFN (IFNλ) 38, 145. Induction of BST‐2 by IFNs occur in a broad range of cell lines, primary cells, and in vivo 4, 8, 28, 38, 45. The effect of IFNs is cell type dependent. In some cells, IFNα is a better inducer of BST‐2. However, in hepatocytes, IFNγ and IFNλ are more potent inducers of BST‐2 38. IFNs from different species are highly conserved among vertebrates 146. IFNs have cross‐species activity on BST‐2 and possibly other IFN‐inducible genes. BST‐2 from one species is responsive to IFNs from another species 147, 148, suggesting that induction of BST‐2 by IFNs may not be an evolutionarily acquired trait. Types I, II, and III IFNs exhibit similar biological and functional activities although they bind to different receptors. Thus, the induction of BST‐2 by all three types of IFNs indicates that multiple signaling pathways regulate BST‐2 expression at least in human hepatocytes 38. Indeed, BST‐2 promoter contains binding elements for STAT1 and STAT3 21, 149. Other sequences present in the promoter region of BST‐2 include that of NF‐κB binding sites 145, AP‐2, and GATA1 21, 149, as well as IL‐6‐responsive elements 21. IFN‐mediated signaling has been used to induce BST‐2 to prevent viral replication and release, as well as an “adjuvant” to enhance the therapeutic potential of anti‐BST‐2 antibodies 38, 121, 139. Contrary to the induction of BST‐2 by IFN, it has been shown that BST‐2 regulates IFNα and IFNγ during CHIKV infection 45 because loss of BST‐2 results in increased viremia and reduced expression of IFNα, IFNγ, and other signaling molecules that are normally increased in CHIKV infected wild‐type mice 45.

BST‐2 is a biological ligand for ILT7 150. Engagement of ILT7 by BST‐2 regulates innate immune functions of pDCs, especially suppression of IFN in an inflammatory environment 150 (Fig. 2 #7). However, in a tumor microenvironment where BST‐2 is constitutively elevated, BST‐2–ILT7 interaction is predicted to suppress pDCs‐mediated normal IFN response to TLR ligands 150. As the interaction between BST‐2 and ILT7 suppress pDCs‐mediated IFN responses required for deterring tumor growth 151, 152, it is tempting to speculate that elevated BST‐2 in tumors 119, 131, 153 and engagement of ILT7 by BST‐2 may contribute to tumor tolerance and progression.

### Cytokine‐mediated BST‐2 regulation

BST‐2 expression is inducible in immune and cancer cells by cytokines. Treatment of monocytes and T cells with IL‐27 induces BST‐2 in an IFN signaling‐independent manner 154. IL‐27 is a cytokine produced by myeloid cells 155 and functions to inhibit HIV‐1 replication in various cell types 156, 157, 158. Whether BST‐2 is one of the effectors of anti‐HIV‐1 activity of IL‐27 is yet to be determined.

In cancer cells, BST‐2 expression is transcriptionally regulated in TGF‐β responsive breast cancer cells 123. Treatment of low grade (grades 1 and 2) breast cancer cell lines with TGF‐β resulted in suppression of BST‐2 transcripts. In contrast, grade 3 cancer cell lines are unresponsive to TGF‐β signaling and BST‐2 expression is not inhibited. Reduction in BST‐2 expression upon TGF‐β treatment correlates with enhanced AP2 binding to the BST‐2 promoter 123. AP2 is a transcription factor involved in repression of promoter sequences of at least one oncogene ERBB2 159. These findings suggest that in breast cancer cells, there is a progressive loss of TGF‐β signaling responsiveness that may result in aberrant BST‐2 overexpression.

## Induction of BST‐2 by Toll‐Like Receptors (TLRs)

TLR‐mediated signaling depends on conserved intracytoplasmic TIR domains. Functionally, TLRs recognize specific but conserved pathogenic components and have been established to play an essential role in the activation of innate immunity, including induction of antiviral factors, such as BST‐2 8. TLRs have been reported to regulate BST‐2 expression and function in different cell types and conditions as discussed below.

### TLR3

In human monocyte derived macrophages (MDM), TLR3 induces the expression of BST‐2 upon infection with HIV‐1. Signaling through TLR3 mediates restriction of virus infection and replication in MDMs 160. This observation was made in macrophages derived from rhesus macaques where TLR3 induces the expression of BST‐2 and other restriction factors 161, 162. Although the elements of TLR3‐mediated induction of BST‐2 are yet to be determined, TLR3 induces BST‐2 expression in peripheral blood mononuclear cells (PBMCs) independent of IFN signaling during early immune responses 145 and treatment of PBMCs with poly(I · C), a TLR3 agonist increased BST‐2 levels 162. BST‐2 promoter contains IRF binding elements and a single IRF binding site renders the BST‐2 promoter responsive to induction by IFNα 145. Additionally, expression of IRF‐1 or virus‐activated forms of IRF‐3 and IRF‐7 activates BST‐2 promoter in the absence of type I IFN signaling 145. Moreover, vesicular stomatitis virus induces BST‐2 in infected mouse embryonic fibroblasts in an IRF‐3/IRF‐7 dependent but type I IFN‐independent pathway 145.

### TLR4

Accumulating evidence indicates that TLR4 has both positive and negative regulatory roles on BST‐2. Ligand activation of TLR4 elicits various signaling pathways including the phosphatidylinositol 3‐kinase (PI3K)/serine/threonine‐specific protein kinase (AKT) pathway 163, 164. In macrophages, TLR4 activation induces BST‐2 expression through a pathway dependent on TRIF and IRF3 signaling 8. Jones et al., found a positive regulatory role for TRIF and IRF3 because deletion of TRIF and IRF3 and pharmacological inhibition of the interactions of TLR4 with TIRAP and TRAM abrogating LPS‐mediated induction of BST‐2 in macrophages 8. Surprisingly, the Myd88 and PI3K pathway results in suppression of BST‐2 expression in macrophages 8 (Fig. 2 #3). TLR4 and PI3K transcriptionally regulate BST‐2 expression given that blockade of BST‐2 transcription with actinomycin D (Act D) disrupts BST‐2 mRNA stability. These observations from Jones et al., highlight the ability of the host to tightly control BST‐2 in normal and inflammatory conditions, especially during viral infection. Indeed, during cis‐infection of HIV‐1 in the viral synapses between immature dendritic cells and CD4+ T cells, TLR4 induces BST‐2 expression and prevents HIV‐1 dissemination across viral synapses 165.

### TLR7/9

Mammalian TLR7 and TLR9 are endosomal sensors of microbial and self‐RNA or DNA, respectively 166, 167, 168, 169. Stimulation of TLR7 or TLR9 by nucleic acids in relevant cell types triggers signal transduction cascades that result in secretion of inflammatory molecules including type I IFNs 169, 170, 171. In PBMCs and not CD4+ T cells, activation of TLR9 with the agonist ODN2216 (type A CpG DNA) induces BST‐2 expression 162. The lack of BST‐2 induction in CD4+ T cells was attributed to absence of TLR9 expression 162. On the other hand, the relationship between TLR7/9 and BST‐2 in pDCs is one of a negative regulation 150, 172, that may result in manifestation of diseases, such as lupus and cancer 122, 173.

## Epigenetic Regulation of BST‐2 Expression

Epigenetic regulation of gene expression is a stable modification in gene expression and function without alterations in DNA sequence. Recently, in silico analyses of the BST‐2 gene demonstrates that BST‐2 expression is epigenetically regulated and that dysregulation of BST‐2 epigenetic landscape may have pathological consequences 119, 173. BST2 expression is inversely proportional to the methylation status of CpGs located inside and in proximity to its promoter region in human breast tumors and in breast cancer cell lines 119. Importantly, highly invasive cancer cells with elevated BST‐2 are hypomethylated while luminal breast cancer cells which are mostly noninvasive are low in BST‐2 and are hypermethylated 119. This pattern of BST‐2 demethylation in breast cancer may be important for cancer cells to acquire an invasive potential. Regulation of BST‐2 expression by CpG methylation has been reported in other disease conditions. In lupus, an autoimmune disease 173 and cervical cancer 124, BST‐2 is hypomethylated and BST‐2 expression is significantly elevated in comparison to control specimens.

## BST‐2 Regulation by Non‐Coding RNAs

RNAseq analysis identified a long non‐protein‐coding RNAs named BISPR (BST2 IFN‐stimulated positive regulator) as a positive regulator of BST‐2 in IFNα2‐treated hepatocellular carcinoma (Huh7) cells. BISPR is expressed from same promoter as BST‐2 but on the opposite direction and its transcription precedes that of BST‐2 174, 175. BISPR and BST‐2 are correlatively upregulated and post‐transcriptional inhibition of BISPR results in reductions in BST‐2 mRNA levels 174. Mutant HCV, influenza, and VSV viruses that are able to activate IFN response induce BISPR and BST‐2 in infected cells, suggesting a functional role for BISPR 174.

## Regulation of BST‐2 by Oncogenic Viruses

Studies from the Ross Lab have been instrumental in deciphering the roles of restriction factors and other host proteins in Mouse mammary tumor virus (MMTV)‐induced mammary oncogenesis 176, 177, 178, 179, 180. Sequences similar to MMTV are present in human breast tumors 181, 182, 183 and cultures of human breast cancer cells produce human mammary tumor virus (HMTV) with morphologic and molecular characteristics of MMTV and with 95% homology with MMTV 184. MMTV promotes breast tumor formation following oncogene activation by integrated provirus into breast epithelial cells 185 and inhibition of epithelial cell apoptosis 186. Similar to the observation in human breast tumors, levels of BST‐2 in MMTV‐induced tumors was significantly elevated 153.

During MMTV infection, BST‐2 restricts MMTV release and replication 4, but once infection is established, MMTV dysregulates BST‐2 expression in a tissue‐specific manner 153. In immune cells of MMTV infected mice, BST‐2 expression is first upregulated and then significantly downregulated. Although the initial increase in immune cells BST‐2 levels may trigger immune response to infection, the down regulation of BST‐2 in these cells may be a mechanism of optimal virus release for efficient infection of distal targets such as the mammary gland 153. Surprisingly, BST‐2 expression is elevated in mammary and tumor tissues 153. Elevated tumor‐associated BST‐2 in mice 153 is in agreement with high BST‐2 levels in human breast tumors 119, 131. Thus, it is possible that MMTV infection of mammary epithelial cells leads to accumulation of epigenetic aberrations that change BST‐2 levels and affect the activity of cancer‐promoting pathways.

MMTV‐mediated dysregulation of BST‐2 in murine mammary tissues is not attributable to IFN since levels of IFNα and IFNγ negatively correlate with BST‐2 153. Nonetheless, soluble factors released by mammary tumor cells suppress IFNα and IFNγ but induce BST‐2 expression 153. These data indicate that overexpression of BST‐2 in carcinoma tissues, at least in this infective model cannot be attributed to IFNs but to factors that upregulates BST‐2 once oncogenesis is initiated.

## Conclusions and Perspectives

**Figure 4:**
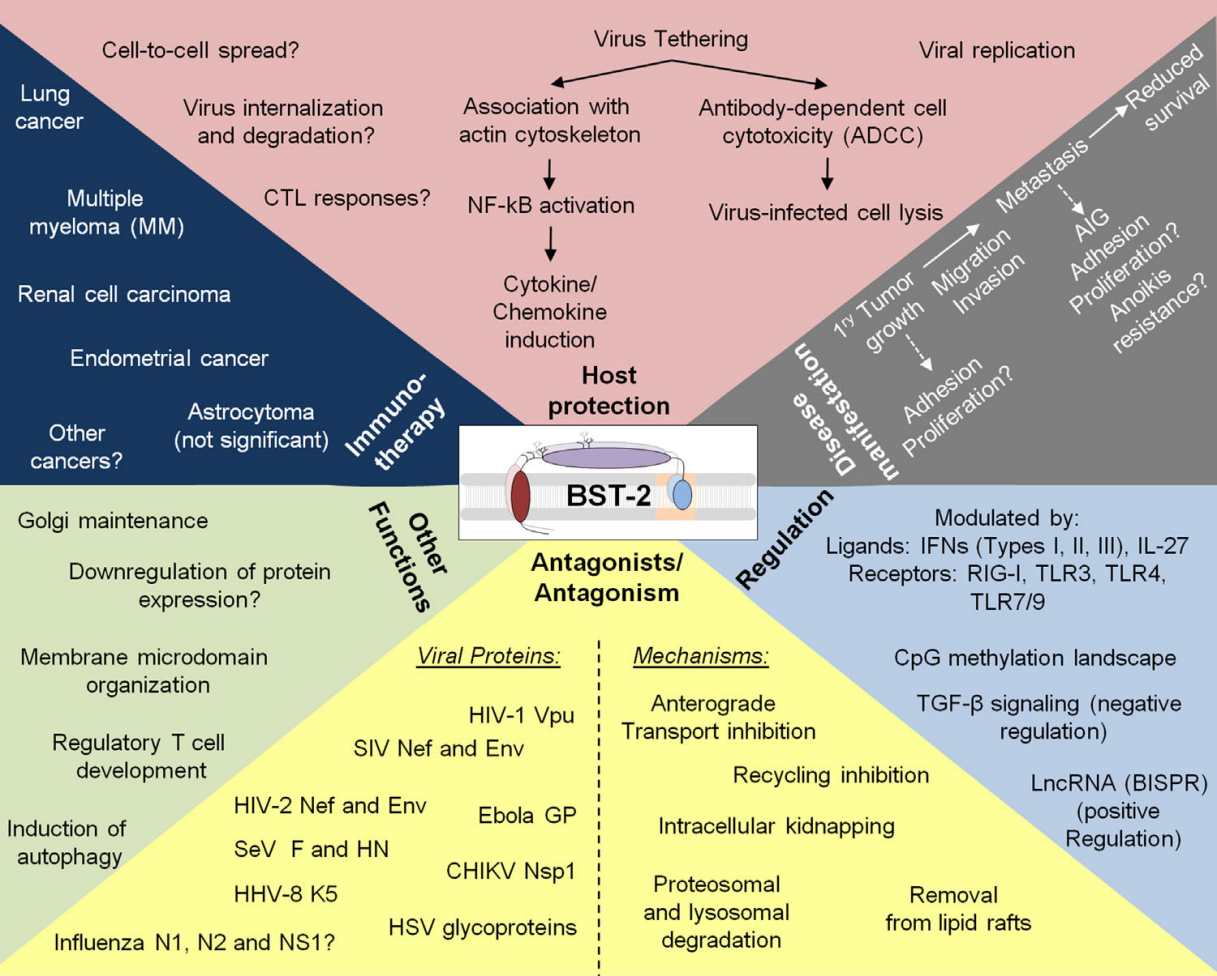
The “moonlight” protein BST‐2: BST‐2 is a protein with several functions that spans the fields of virology, immunology, and cancer biology. The most defining antiviral function of BST‐2 is tethering of enveloped viruses to the surface of infected cells. Virus tethering results in stimulation of the immune system, prevention, or enhancement of viral replication and spread, as well as initiation of ADCC (pink panel). Viruses from different families have the capacity to antagonize BST‐2 by means of their accessory proteins. These include HIV‐1 Vpu, HIV‐2 Env, SIV Nef, and HHV‐8 K5 or through their structural and nonstructural proteins, such as Ebola GP, CHIKV nsP1, and HSV‐2 glycoproteins (yellow panel, left). The mechanisms used by various viral proteins to neutralize BST‐2 effect include, but not limited to degradation via the proteasomal and lysosomal pathways, removal from lipid rafts, inhibition of anterograde transport, intracellular trapping (in endosomes, ER and Golgi) and unknown mechanisms, all of which result in reduced BST‐2 surface levels (yellow panel, right). Furthermore, BST‐2 plays a cell type dependent role in Golgi maintenance, downregulation of viral protein expression, organization of membrane microdomains, regulation of Treg development, and induction of autophagy (light green panel). The role of BST‐2 in promoting cell‐to‐cell interaction, cell‐to‐ECM interaction, and enhancement of disease is highlighted. Through significant enhancement of cellular behaviors, BST‐2 promotes primary tumor growth and metastasis resulting in poor clinical outcomes (gray panel). Therapeutically, BST‐2 ADCC function has been exploited in the treatment of lung cancer, multiple myeloma, renal cell carcinoma, and endometrial cancer. Anti‐HM1.24 treatment of astrocytoma was not of therapeutic significance. This treatment may be useful for other high BST‐2 expressing cancers (dark blue panel). Ligands, such as IFNs (interferons) and ILs (interleukins) or receptors including IFNR, nucleic acid‐sensing pattern recognition receptors RIG‐I (retinoic acid‐inducible gene 1), and TLRs (toll‐like receptors) regulate BST‐2. A long non‐coding RNA named BST‐2 IFN‐stimulated positive regulator (BISPR) was found to induce the expression of BST‐2. In cancer, BST‐2 expression is epigenetically regulated by hypomethylation of specific CpGs, by TGF‐β signaling, and by yet to identified mechanisms during viral carcinogenesis (unknown).

Emerging experimental and clinical evidence suggest that BST‐2 as a host restriction factor is a moonlight protein (Fig. 4) that is crucial for regulation of cell signaling and maintaining host innate and cellular homeostasis. However, fundamental questions remain relating to how BST‐2 orchestrates multifunctional roles in protection against and manifestation of disease. Although some structural features of BST‐2 are shared between these roles, differences abound. For example, the virus tethering function of BST‐2 may be comparable to its cancer promoting function in cell to cell adhesion but different BST‐2‐induced signals may be required for these two processes. Given that BST‐2 tethers lipid membrane containing viruses and mediates cell to cell adhesion 131, 132 and to ECM proteins 131, it remains to be determined whether BST‐2 can tether other membrane‐containing pathogens and membrane‐containing vesicles, such as bacteria and exosomes. Whether the anti‐viral and pro‐cancer functions of BST‐2 reflect cell or tissue specific differences in levels of BST‐2 is unclear. However, variability in BST‐2 levels has functional importance as high BST‐2 expressing cells are poor producers of cell‐free virus 6, 45 whereas high BST‐2 expressing breast cancer cells are highly invasive both in ex vivo invasion and in vivo animal models 122.

Indeed, the role of BST‐2 in viral pathogenesis, especially HIV‐1 is still unknown as most HIV‐1 experiments have been performed in cultured cells. However, using patient‐derived specimens, it was shown that pandemic HIV‐1 group M express a Vpu variant that antagonizes BST‐2 and CD4 whereas Vpu from non‐pandemic HIV‐1 strains does not antagonize BST‐2 187. Additionally, the neutralizing effect of BST‐2 by Vpu is not absolute in HIV‐1 infected patients 162 and BST‐2 has developed an immune sensing function for HIV‐1 clearance in vivo 41. These data point to the Vpu‐BST‐2 antagonistic interaction as a significant determinant of the ability of either HIV‐1 to promote its spread or of the host to restrict the virus.

In breast cancer, patients bearing high BST‐2‐expressing tumors have poor survival compared to patients bearing low BST‐2‐expressing tumors. Corroboration of this observation in mouse model of breast cancer 131 demonstrates that pathological BST‐2 upregulation in tumors may by itself be sufficient to cause or predict clinical disease, and that inhibiting BST‐2 activity in tumor cells is sufficient to produce good clinical outcome.

The ability of BST‐2 to inhibit virus infection and promote carcinogenesis highlights the need to determine whether the antiviral or protumor function of BST‐2 will dominate in the pathogenesis of oncoviruses or viruses that activate cellular oncogene expression. For example, BST‐2 expression restricts MMTV release and inhibits MMTV replication 4. However, chronic MMTV infection down‐regulates BST‐2 in hematopoietic cells but upregulates BST‐2 in mammary gland and tumor tissues 153. Perhaps, MMTV‐mediated BST‐2 induction and repression in the same host may lead to aberrant BST‐2 regulation, triggering breast oncogenesis. Whether MMTV‐mediated BST‐2 dysregulation is triggered by viral particles or by host responses to MMTV infection is unknown. The tethering and antiviral functions of BST‐2 are intact in transformed breast epithelial cells 4, so is the protumor role of BST‐2 in transformed epithelial cells 131. BST‐2 contains several motifs on its N‐terminal cytoplasmic tail that may be involved in the activation of multiple kinases thereby expanding the signaling capacity of BST‐2 to several intracellular pathways that may impact the way cells respond to viral infections and cancer. Also, some motifs on the BST‐2 cytoplasmic tail may be phosphorylated increasing the complexity and breadth of BST‐2 than previously thought.

The involvement of BST‐2 in viral infection, cancer, and lupus might reflect variable engagement of the host innate and adaptive immune systems regulation and dysregulation under different conditions. The findings that BST‐2 is epigenetically regulated in cancer and autoimmune diseases indicate the possibility of yet‐to‐be discovered BST‐2‐related biological pathways of importance in the context of human disease and treatment. Identification of the cellular triggers that regulate BST‐2 expression and activity in patients infected with BST‐2‐susceptible viruses or in patients bearing BST‐2‐dependent cancers is of fundamental importance. Evaluation of these triggers and identification of their targets will provide the much needed tools for therapeutic manipulation of BST‐2 and BST‐2 signal transduction pathways. Considering the complexity of BST‐2 expression and its functions, we predict the identification of more diseases in which constitutive or induced BST‐2 expression or signaling will control, thus broadening the roles of BST‐2 in protection against or manifestation of disease.

## Conflicts of Interest

The authors declare no conflicts of interest.
